# A novel device with pedicular anchorage provides better biomechanical properties than balloon kyphoplasty for the treatment of vertebral compression fractures

**DOI:** 10.1186/s40634-023-00635-7

**Published:** 2023-07-21

**Authors:** Jean-Charles Le Huec, Thomas Droulout, Lisa Boue, Edouard Dejour, Sonia Ramos-Pascual, Stephane Bourret

**Affiliations:** 1 Polyclinique Bordeaux Nord Aquitaine, Vertebra Center, 33 Rue du Dr Finlay, 33300 Bordeaux, France; 2 Safe Orthopaedics, Allée Rosa Luxemburg, 95610 Eragny Sur Oise, France; 3 ReSurg SA, Rue Saint-Jean 22, 1260 Nyon, Switzerland

**Keywords:** Vertebral compression fracture, dowelplasty, Biomechanical study, Balloon kyphoplasty, Fracture load

## Abstract

**Purpose:**

To compare the biomechanical behavior of vertebrae with vertebral compression fractures (VCF) treated by a novel system with pedicular anchorage (dowelplasty) versus balloon kyphoplasty.

**Methods:**

Four cadaveric spines (T12-L5) were harvested, cleaned from soft tissues, and separated into vertebrae. Axial compressive loads were applied to each vertebra until a VCF was generated. Half of the vertebrae (*n* = 11) were instrumented using the “dowelplasty” system, consisting of a hollow titanium dowel anchored into the pedicle, through which a cannulated titanium nail is inserted and locked and through which cement is injected. The other half (*n* = 11) were instrumented using balloon kyphoplasty. Axial compressive loads were re-applied to each vertebra until fracture. Fracture load and fracture energy were calculated from load–displacement data for the pre- and post-treatment states.

**Results:**

Compared to balloon kyphoplasty, dowelplasty granted greater net change in fracture load (373N; 95%CI,-331–1076N) and fracture energy (755Nmm; 95%CI,-563–2072Nmm). A sensitivity analysis was performed without L4 and L5 vertebrae from the dowelplasty group, since the length of the cannulated nails was too short for these vertebrae: compared to balloon kyphoplasty, dowelplasty granted an even greater net change in fracture load (680N; 95%CI,-96–1457N) and fracture energy (1274Nmm; 95%CI,-233–2781Nmm).

**Conclusion:**

Treating VCFs with dowelplasty grants increased fracture load and fracture energy compared to the pre-treatment state. Furthermore, dowelplasty grants greater improvement in fracture load and fracture energy compared to balloon kyphoplasty, which suggests that dowelplasty may be a good alternative for the treatment of VCF.

**Level of evidence:**

level IV.

## Introduction

Worldwide, 1.4 million cases of vertebral compression fractures (VCFs) are estimated to occur each year [9]. Percutaneous vertebroplasty (PVP) has traditionally been performed to treat VCF; however, guidelines from the American Academy of Orthopedic Surgeons strongly recommend against its use, due to its uncertain benefits and known harms [17]; instead, other surgical treatments are now commonly performed on their own or in combination with cement injection, including percutaneous balloon kyphoplasty (PBKP) and percutaneous vertebral augmentation systems (PVAS) [2, 5, 8, 11]. These surgical treatments intend to restore and maintain vertebral body height and reduce kyphosis, which in turn decreases low back pain and reduces the risk of mortality [2, 10].

A number of studies have found no clinically relevant differences between PVP, PBKP, and PVAS in their ability to restore vertebral body height and reduce kyphosis, and maintain it in the long-term [4, 7, 12, 16]. Furthermore, a recent meta-analysis [5] stated that it is unclear whether PVAS is superior to PBKP or PVP in terms of pain relief and functional improvement. Korovessis et al. [12] compared anterior vertebral body height preoperatively versus at 13–15 months follow-up for PBKP and PVAS, and found an increase of only 23% and 24% respectively, with no significant differences between treatment groups. In addition, Li et al. [15] compared anterior vertebral body height immediately after surgery versus at 12 months follow-up for PBKP, and found a decrease of more than 65%. These findings suggest that the available surgical treatments for VCF are not able to provide the necessary biomechanical strength to maintain vertebral body height.

**Fig. 1:**
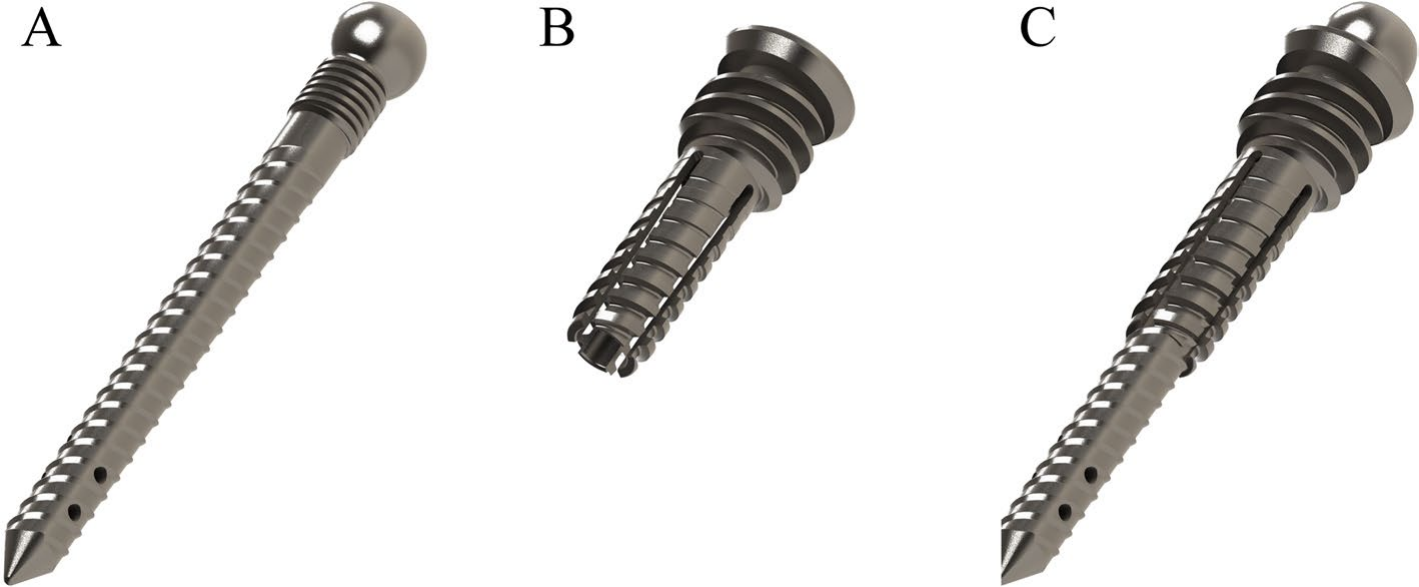
The dowelplasty system (Sycamore, Safe Orthopaedics, Eragny sur Oise, France) consists of (**a**) a cannulated titanium nail, and (**b**) a hollow titanium dowel that is directly anchored into the pedicle; **c** the cannulated nail is inserted and locked into the hollow dowel, and cement is injected through it

A new system, named dowelplasty, has been developed to treat VCF that consists of a cannulated titanium nail through which cement is injected, the nail is in turn inserted and locked into a hollow titanium dowel that is directly anchored into the pedicle (Fig. 1). Dowelplasty may provide increased biomechanical strength compared to other devices or treatments, as the dowel is anchored mechanically into the pedicle. The purpose of the present study was to compare the biomechanical behavior of a vertebra with a VCF treated by dowelplasty versus balloon kyphoplasty. The null hypothesis was that the mechanical behavior would be similar regardless of the treatment.

## Materials and methods

### Specimen preparation

Four freshly frozen human cadaveric spines (T12-L5) were harvested from four females, aged 71, 83, 86, and 89 years old, with a T-score of 4.3, 3.2, 3.8, and 3.8 respectively, determined by dual-energy X-ray absorptiometry (DEXA). Inclusion criterion for the samples were their availability from the university’s anatomic laboratory. Exclusion criteria were (i) T-score < 2.5 determined by DEXA, as this would not correspond with osteoporotic bone, or (ii) a pedicle < 6 mm, as the cannulated nail that is part of the dowelplasty system has a diameter of 6 mm. All soft tissues, including ligaments, were removed from the thoracolumbar spines, and adjacent vertebrae were then separated. Each spine produced 6 specimens, resulting in a total of 24 specimens. Superior and inferior endplates were cleaned of any remaining disc tissue and potted using Wood’s metal (low melting-point alloy), ensuring the endplates were parallel to the pots, so that the endplate would be normal to the applied axial load. After potting, each specimen was wrapped in saline-soaked gauze and cling film and frozen until the day of fracture creation.

### Fracture generation

Specimens were mounted on a displacement-controlled testing machine, and an axial compressive preload of 20N was applied at the anterior third of the vertebral body. The axial compressive load was increased in steps of 0.25 mm until a vertebral fracture was generated, which was defined as maximum peak load, followed by no load resistance. Loads and displacements were saved and exported. Specimens were removed from the testing machine and radiographic analysis was performed to confirm fracture generation. Specimens were then wrapped in saline-soaked gauze and cling film and stored at 4ºC until the day of surgical treatment.

### Surgical treatment

Specimens were evenly distributed into the dowelplasty and balloon kyphoplasty groups (twelve specimens per group), according to the level of the spine (Table 1). All procedures were performed by an experienced spine surgeon (JCLH) under fluoroscopic control, using dedicated instrumentation kits.

**Table 1:** Characteristics of the included cadavers

Donor	Sex	Age	T-score	Treatment groups (treated levels)
Spine 1	F	83	3.2	Dowel: T12, L2, L4 Balloon kyphoplasty: L1, L3, L5
Spine 2	F	86	3.8	Dowel: L1, L3, L5 Balloon kyphoplasty: T12, L2, L4
Spine 3	F	89	3.8	Dowel: T12, L2, L4 Balloon kyphoplasty: L1, L3, L5
Spine 4	F	71	4.3	Dowel: L2, L4 Balloon kyphoplasty: L3, L5

**Fig. 2:**
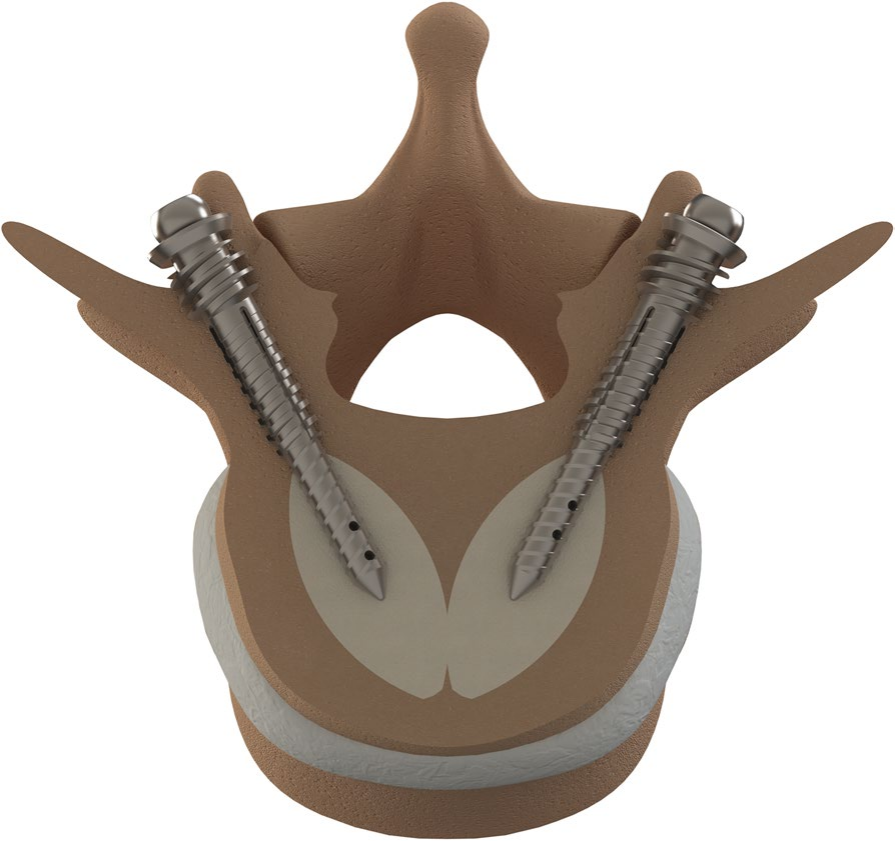
The dowelplasty system (Sycamore, Safe Orthopaedics, Eragny sur Oise, France) implanted into a vertebra

The test group was instrumented using a dowelplasty system (Sycamore, Safe Orthopaedics, Eragny sur Oise, France). First, trocars were inserted into each pedicle, then guidewires were placed inside the vertebral body using Jamshidi needles, and the trocars were removed. A manual bone drill was then used to enlarge each pedicle hole, after which a hollow dowel was inserted through each pedicle. A balloon (SteriSpine VA, Safe Orthopaedics, Eragny sur Oise, France) was inserted through each dowel and inflated with dye to restore height and reduce kyphosis. After balloon removal, cannulated nails were inserted and locked into each dowel, and cement was injected through each cannulated nail (Fig. 2). The same size of dowel (6 mm diameter and 25 mm length) and cannulated nail (5 mm diameter and 40 mm length) were used for all specimens. Different length nails are produced by the manufacturer, but were not available on the day of surgical treatment.

The control group was instrumented using balloon kyphoplasty (SteriSpine VA, Safe Orthopaedics, Eragny sur Oise, France). First, trocars were inserted into each pedicle, then guidewires were placed inside the vertebral body using Jamshidi needles, and then working cannulas were placed using the guidewires, after which the guidewires were removed. A manual bone drill was then used to enlarge each pedicle hole. Then, a balloon was inserted through each trocar and inflated with dye to restore height and reduce kyphosis. After balloon removal, cement was injected through each trocar.

### Cement

The same cement was used for both groups (Safe Orthopaedics, Eragny sur Oise, France). Cement injection volume was 5 ml for all specimens, this volume was chosen as it was sufficient to restore the vertebral shape without filling the vertebra with cement. Following injection, cement was allowed to cure for 15 min at room temperature.

### Biomechanical testing following surgical treatment

Two of the 24 specimens (one from each group) had to be excluded following surgical treatment, because (i) one did not reach the correct cement polymerization time, and (ii) the other had a pedicle that was too small for device implantation.

Specimens were re-mounted on the displacement-controlled testing machine, and an axial compressive preload of 20N was applied at the anterior third of the vertebral body. The axial compressive load was increased in steps of 0.25 mm until a vertebral fracture was generated, which was defined as maximum peak load, followed by no load resistance. Loads and displacements were saved and exported. Specimens were removed from the testing machine and radiographic analysis was performed to confirm the integrity of the pedicle.

### Data analysis

From the load and displacement values, the fracture load and fracture displacement were identified for each specimen. Furthermore, energy absorbed at fracture was calculated by multiplying the fracture load and fracture displacement, while stiffness was taken to be the slope of a linear model fitted to the load–displacement curve that was forced to have a y-intercept of zero. Descriptive statistics were used to summarise the data. Differences between pre- and post-treatment values were calculated as net changes. Effect sizes were calculated as mean differences between treatment groups with their 95% confidence intervals (CI). Since there were less than 30 samples, data was assumed to be non-normally distributed [6]. For paired data (pre- vs post-treatment), comparisons between groups were performed using Wilcoxon signed rank tests. For unpaired data (dowelplasty vs balloon kyphoplasty), comparisons between groups were performed using Wilcoxon rank sum tests. Statistical analyses were conducted using R version 3.6.1 (R Foundation for Statistical Computing). *P*-values < 0.05 were considered statistically significant.

## Results

**Fig. 3:**
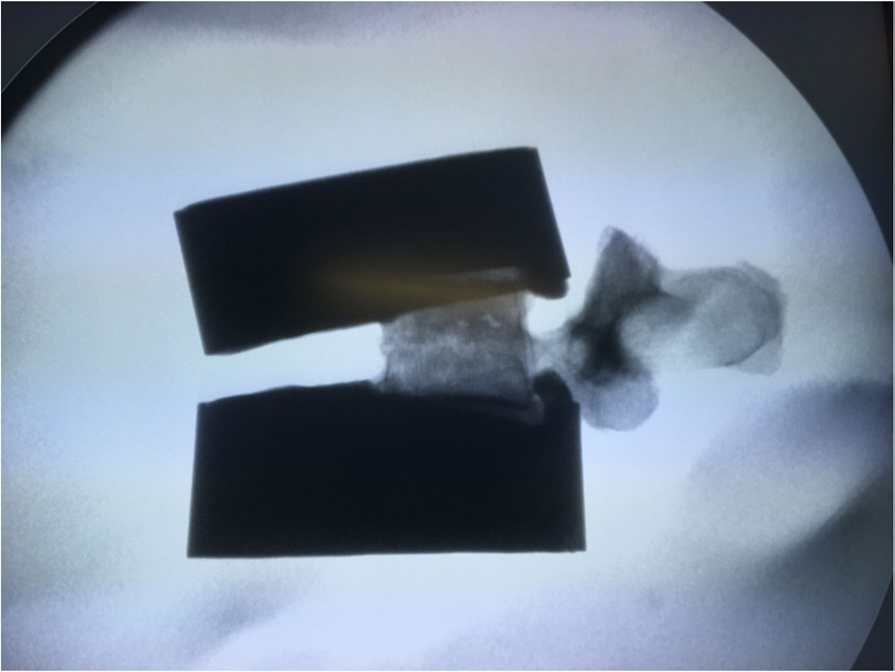
Example of radiograph after fracture generation, which shows a vertebral compression fracture

**Fig. 4:**
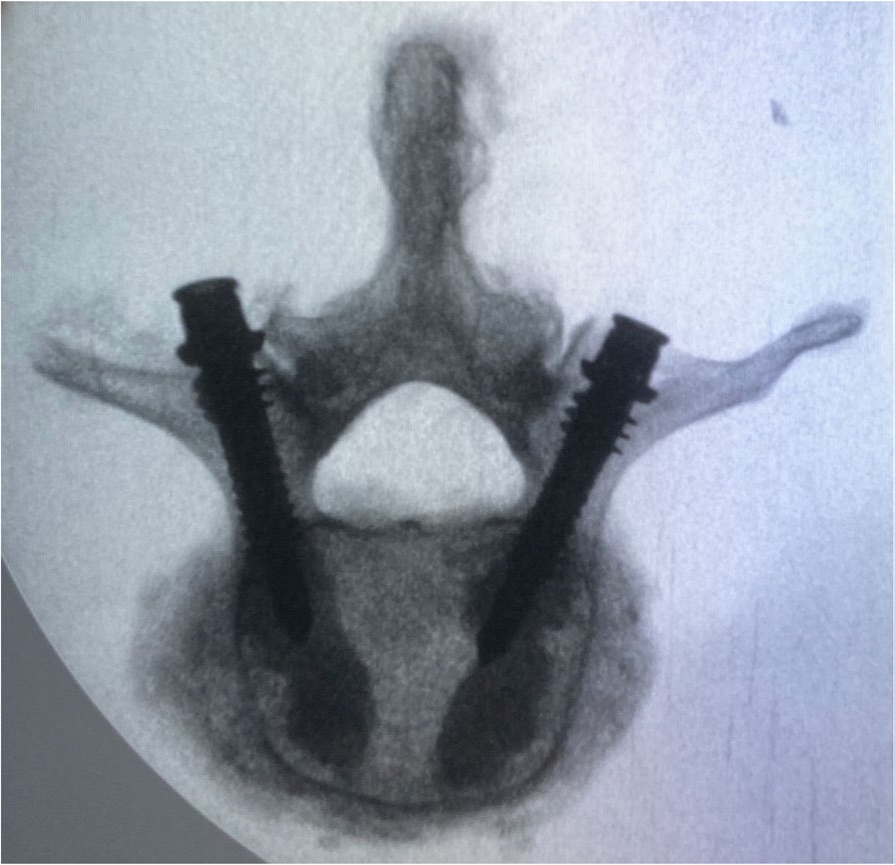
Example of radiograph after surgical treatment and biomechanical testing, which shows pedicle integrity

Radiographic analysis after fracture generation confirmed consistent vertebral fractures were produced in all specimens (Fig. 3), while radiographic analysis after surgical treatment and biomechanical testing confirmed the integrity of the pedicle in all specimens, as well as the integrity of the implant in the dowelplasty group. The length of the cannulated nails (40 mm) used for the specimens in the dowelplasty group were found to be insufficient for the L4 and L5 vertebral bodies, as they reached < 75% of their width (Fig. 4).

**Fig. 5:**
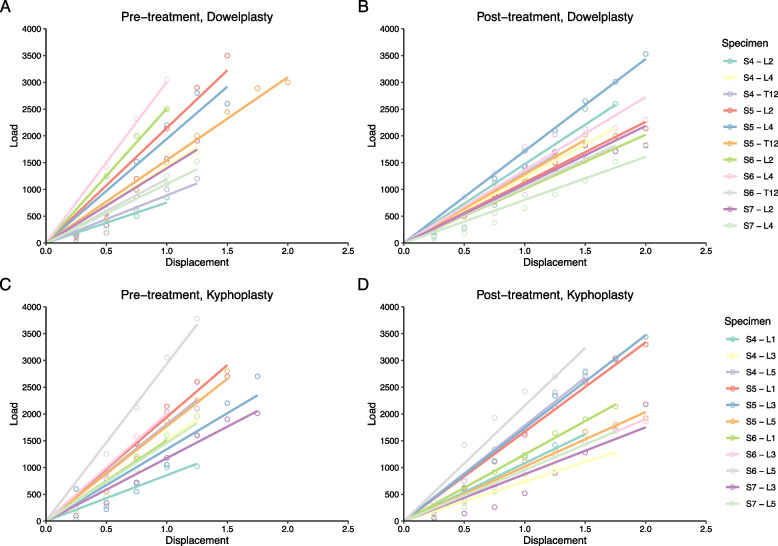
Load–displacement curves for the dowelplasty group (**a**) pre-treatment and (**b**) post-treatment, as well as for the balloon kyphoplasty group (**c**) pre-treatment and (**d**) post-treatment

The mean fracture load increased between pre- and post-treatments for the dowelplasty group (2065 ± 854N to 2291 ± 648N), while it decreased for the balloon kyphoplasty group (2256 ± 775N to 2109 ± 612N) (Table 2). The mean fracture energy increased between pre- and post-treatments for both the dowelplasty group (2729 ± 1416Nmm to 4199 ± 1421Nmm) and the balloon kyphoplasty group (3104 ± 1502Nmm to 3819 ± 1343Nmm). The mean stiffness decreased between pre- and post-treatments for both the dowelplasty group (1552 ± 607N/mm to 1335 ± 396N/mm) and the balloon kyphoplasty group (1743 ± 613N/mm to 1180 ± 332N/mm) (Fig. 5).

**Table 2:** Failure load, energy at fracture, and stiffness, stratified by treatment group

	**Dowelplasty (*n*=11)**		**Balloon kyphoplasty (*n* = 11)**		**Mean difference**	**(95%CI)**	*p*-value**
	**Mean ± SD**	**(95%CI)**	**Mean ± SD**	**(95%CI)**			
**Fracture load**							
Pre-treatment (N)	2065 ± 854	(1492 –2639)	2256 ± 775	(1736 –2777)	-191	(-916 –534)	0.511
Post-treatment (N)	2291 ± 648	(1855 –2726)	2109 ± 612	(1698 –2520)	182	(-379 –743)	0.743
Net change (N)	225 ± 765	(-289 –740)	-147 ± 815	(-695 –400)	373	(-331 –1076)	0.375
*p*-value*	0.365		0.520				
**Fracture energy**							
Pre-treatment (Nmm)	2729 ± 1416	(1778 –3680)	3104 ± 1502	(2095 –4113)	-375	(-1673 –924)	0.430
Post-treatment (Nmm)	4199 ± 1421	(3245 –5154)	3819 ± 1343	(2917 –4722)	380	(-850 –1610)	0.577
Net change (Nmm)	1470 ± 1260	(624 –2317)	716 ± 1673	(-408 –1840)	755	(-563 –2072)	0.270
*p*-value*	0.007		0.123				
**Stiffness**							
Pre-treatment (N/mm)	1552 ± 607	(1144 –1960)	1743 ± 631	(1319 –2166)	-190	(-741 –360)	0.365
Post-treatment (N/mm)	1335 ± 396	(1069 –1601)	1180 ± 332	(957 –1403)	155	(-170 –480)	0.401
Net change (N/mm)	-217 ± 546	(-584 –150)	-562 ± 604	(-968 – -156)	345	(-167 –857)	0.401
*p*-value*	0.320		0.005				

Abbreviations: *SD* Standard deviation, *CI* Confidence interval

^*^ *P*-values comparing pre- versus post-treatment using paired wilcoxon signed rank tests

^**^ *P*-values comparing dowelplasty versus kyphoplasty using unpaired wilcoxon rank sum tests

Compared to the balloon kyphoplasty group, the dowelplasty group granted a post-treatment fracture load that was 182N greater (95%CI, -379–743N), a net change in fracture load that was 373N greater (95%CI, -331–1076N), a post-treatment fracture energy that was 380Nmm greater (95%CI, -850–1610Nmm), a net change in fracture energy that was 755Nmm greater (95%CI, -563–2072Nmm), a post-treatment stiffness that was 155N/mm greater (95%CI, -170–480N/mm), and a net change in stiffness that was 345N/mm smaller (95%CI, -167–857Nmm) (Table 2). There were no significant differences in the net difference in fracture load, fracture energy, and stiffness between the dowelplasty and balloon kyphoplasty groups, probably because of the small sample size.

A sensitivity analysis was performed removing the L4 and L5 vertebrae from the dowelplasty group, as the cannulated nails used during surgery had a length that was insufficient for the vertebral bodies. Compared to the balloon kyphoplasty group, the dowelplasty group granted a post-treatment fracture load that was 271N greater (95%CI, -416–958N), a net change in fracture load that was 680N greater (95%CI, -96–1457N), a post-treatment fracture energy that was 611Nmm greater (95%CI, -946–2167Nmm), a net change in fracture energy that was 1274Nmm greater (95%CI, -233–2781Nmm), a post-treatment stiffness that was 174N/mm greater (95%CI, -150–499N/mm), and a net change in stiffness that was 512N/mm smaller (95%CI, -106–1130Nmm) (Table 3). There were no significant differences in the net difference in fracture load, fracture energy, and stiffness between the dowelplasty and balloon kyphoplasty groups, probably because of the small sample size.

**Table 3:** Sensitivity analysis excluding L4 and L5 vertebrae in the dowelplasty group

	**Dowelplasty (*n*=7)**		**Balloon kyphoplasty (*n*=11)**		**Mean difference**	**(95%CI)**	*p*-value**
	**Mean ±SD**	**(95%CI)**	**Mean ±SD**	**(95%CI)**			
**Fracture load**							
Pre-treatment (N)	1847 ± 712	(850 –2700)	2256 ± 775	(1736 –2777)	-409	(-1180 –362)	0.319
Post-treatment (N)	2380 ± 757	(1500 –3440)	2109 ± 612	(1698 –2520)	271	(-416 –958)	0.650
Net change (N)	533 ± 650	(-210 –1750)	-147 ± 815	(-695 –400)	680	(-96 –1457)	0.113
*p*-value*	0.078		0.520				
**Fracture energy**							
Pre-treatment (Nmm)	2440 ± 1429	(850 –4725)	3104 ± 1502	(2095 –4113)	-663	(-2175 –848)	0.277
Post-treatment (Nmm)	4430 ± 1773	(1875 –6880)	3819 ± 1343	(2917 –4722)	611	(-946 –2167)	0.497
Net change (Nmm)	1990 ± 1049	(375 –3700)	716 ± 1673	(-408 –1840)	1274	(-233 –2781)	0.085
*p*-value*	0.016		0.123				
**Stiffness**							
Pre-treatment (N/mm)	1404 ± 474	(755 –2004)	1743 ± 631	(1319 –2166)	-338	(-930 –254)	0.285
Post-treatment (N/mm)	1354 ± 289	(953 –1736)	1180 ± 332	(957 –1403)	174	(-150 –499)	0.285
Net change (N/mm)	-50 ± 601	(-1051 –719)	-562 ± 604	(-968 – -156)	512	-106 –1130)	0.211
* p*-value*	0.938		0.005				

Abbreviations: *SD* Standard deviation, *CI* Confidence interval

*P-values comparing pre- versus post-treatment using paired wilcoxon signed rank tests

**P-values comparing dowelplasty versus kyphoplasty using unpaired wilcoxon rank sum tests

## Discussion

The most important findings of the present study are that (i) treating a VCF using the dowelplasty system grants an increased fracture load and fracture energy compared to the pre-treatment (native) state, and (ii) dowelplasty grants greater improvement in fracture load and fracture energy compared to balloon kyphoplasty, and provides a smaller change in the vertebra’s stiffness, which suggests that dowelplasty may be a good alternative for the treatment of VCF and may be able to maintain the vertebral shape more effectively than other treatments. It is important to note that, although differences between groups were clinically relevant, this study was underpowered to detect significant differences.

The present study included 24 specimens, of which two had to be excluded during specimen preparation, thus testing was performed on 11 specimens per group. Furthermore, a sensitivity analysis was performed, removing the four L4 and L5 vertebrae in the dowelplasty group, as the cannulated nails used during surgery had a length that was insufficient for the vertebral bodies. This resulted in 7 versus 11 specimens in the dowelplasty and balloon kyphoplasty groups respectively. A post-hoc power analysis indicated a statistical power of 57%, which is inadequate to identify significant differences between groups. Therefore, due to the small sample size, the present study has a high risk of type II error: there may be differences between groups, but these do not appear as significant. Nonetheless, the sensitivity analysis found mean differences between groups of 680N (95%CI, -96–1457N) for net change in fracture load, 1274Nmm (95%CI, -233–2781Nmm) for net change in fracture energy, and 512N/mm (95%CI, -106–1130Nmm) for net change in stiffness, indicating a clinical relevance. It is interesting to note that when including the L4 and L5 vertebrae in the dowelplasty group, there were still considerable mean differences between groups for the net change in fracture load (373N; 95%CI, -331–1076N), net change in fracture energy (755Nmm; 95%CI, -563–2072Nmm), and net change in stiffness (345N/mm; 95%CI, -167–857Nmm). These findings suggest that treating a VCF using the dowelplasty system, even with a cannulated nail that is too short for the vertebral body, provides better outcomes than balloon kyphoplasty.

A number of published cadaveric studies have compared biomechanical outcomes of VCF treated using novel systems versus balloon kyphoplasty or vertebroplasty [1, 3, 18–21], with most studies finding no statistically significant or clinically relevant differences in fracture load, fracture energy, and stiffness between treatment groups. Aebi et al. [1] compared a novel transpedicular implant (hollow cannulated strut through which cement was injected) to vertebroplasty, and reported an increase in fracture load of 68% vs 53%, an increase in fracture energy of 124% vs 131%, and a decrease in stiffness of -50% vs -66%. Rotter et al. [19] compared a novel vertebral augmentation system (stent with cement) to balloon kyphoplasty, and reported an increase in fracture load of 82% vs 64% (*p* = 0.592) and a decrease in stiffness of 21% vs 16% (*p* = 0.862). Wang et al. [21] compared a novel vertebral augmentation system (stent with cement) to balloon kyphoplasty, and reported an increase in fracture load of 89% vs 46% (*p* < 0.05) and a decrease in stiffness of 32% vs 40% (*p* > 0.05). In addition, Upasani et al. [20] compared a novel vertebral augmentation system (titanium stent with cement) to balloon kyphoplasty, and reported a negligible decrease in fracture load of 0% vs 2% (*p* > 0.05) and a decrease in stiffness of 38% vs 40% (*p* > 0.05). In contrast to all but one of the above-mentioned studies, the present study found considerably greater differences between the test (dowelplasty) and control (balloon kyphoplasty) groups, reporting a change in fracture load of + 29% vs -6.5%, an increase in fracture energy of 82% vs 23%, and a decrease in stiffness of 3% vs 32%.

The dowelplasty system evaluated in the present study consists of a hollow titanium dowel directly anchored into the pedicle, through which a cannulated titanium nail is inserted and locked, and through which, in turn, cement is injected. This design acts as a structural beam fixed into the pedicle, providing increased resistance to axial load compared to balloon kyphoplasty, but without considerably changing the stiffness of the vertebra. The structural beam distributes axial load from the weaker cancellous bone of the vertebral body to the posterior processes via the pedicle. Furthermore, the cannulated nail maintains the cement in place, reducing the risk of cement subsidence that can occur with balloon kyphoplasty. Therefore, the dowelplasty system would be expected to better maintain anterior vertebral height and vertebral shape, and therefore should decrease the risk of patient injury compared to balloon kyphoplasty. There is only one other previous study that has evaluated a similar system: Aebi et al. [1] studied a hollow cannulated strut made of polyetheretherketone (PEEK) through which cement was injected. However, an important difference between the two systems is that the hollow strut evaluated by Aebi et al. was not directly anchored to the pedicle.

The present study injected the same amount of cement (5 ml) into all specimens, regardless their treatment group and vertebral level. Cement is known to increase the strength of the vertebra and decrease its stiffness; therefore, by using the same amount of cement, the authors could test the biomechanical properties of the system itself, instead of that of cement. Nonetheless, in clinical practice, surgeons inject the optimal amount of cement, which varies from patient to patient. Previous cadaveric studies reporting biomechanical outcomes of VCF following surgical treatment have used varying amounts of cement volumes, with means ranging between 1.6–8.1 ml [1, 3, 11, 13, 14, 20, 21].

This cadaveric study has a number of limitations. First, the four harvested spines came from women, as there is a 4:1 proportion of donations from women at the university’s anatomic laboratory where the cadavers were acquired. However, gender should not affect the biomechanical results presented, as patients were only included in the study if they had osteoporotic bone, with a T-score < 2.5; furthermore, as this is a comparative study, if gender had an effect on the biomechanical properties, it would have been equal across the two groups. Second, the present study tested single vertebrae, on which all soft tissues had been removed, instead of testing longer functional spinal units, which could have resulted in different load distributions across intervertebral discs and facet joints, and may have considerably changed the results. Third, an a priori sample size calculation was not performed, instead the number of specimens was chosen based on similar previously published studies [1, 13, 14, 21]; it is possible that due to the small sample size, the present study has a high risk of type II error: there may be differences between groups, but these do not appear as significant. Fourth, vertebral body height was not measured for each vertebra in the pre- (native) and post-treatment states, thus it was not possible to evaluate changes in vertebral height as the specimen was tested. Fifth, the volume of cement injection was the same for all specimens. Sixth, the testing machine could only apply displacements in increments of 0.25 mm, instead of continuously, and loads were only recorded every 0.25 mm. Seventh, the length of the cannulated nail was the same for all specimens, which proved to be insufficient for the L4 and L5 vertebral bodies, that would have required a longer nail for optimal performance.

## Conclusions

Treating a VCF using the dowelplasty system grants an increased fracture load and fracture energy compared to the pre-treatment (native) state. Furthermore, dowelplasty grants greater improvement in fracture load and fracture energy compared to balloon kyphoplasty, which suggests that dowelplasty may be a good alternative for the treatment of VCF.

## Data Availability

The datasets used and/or analysed during the current study are available from the corresponding author on reasonable request.
